# The Influence of Connectedness to Nature on Young Children’s Creative Personality: A Parallel Mediation Analysis

**DOI:** 10.3390/bs16071077

**Published:** 2026-07-01

**Authors:** Xiaomin Li, Mengwen Zhang, Wenxuan Li, Hao Zheng, Fengqi Tao, Beijia Tan

**Affiliations:** 1 Henan Province Preschool Education Research Center, Henan University, Kaifeng 475004, China; 2 Department of Early Childhood Education, Henan University, Kaifeng 475004, China; 15517493762@163.com (M.Z.);

**Keywords:** connectedness to nature, creative personality, cognitive flexibility, psychological resilience, children’s creative personality

## Abstract

Objectives: The aim of this study was to examine the relationships among connectedness to nature, cognitive flexibility, psychological resilience, and creative personality in preschool children, and to investigate the parallel mediating roles of cognitive flexibility and psychological resilience in the relationship between connectedness to nature and creative personality. Methods: In this cross-sectional study, 592 preschool children aged 3–6 years and their parents from two kindergartens in K City and N City participated. General sociodemographic data were collected, including place of residence, gender, only-child status, parents’ educational level, and children’s age. The Connectedness to Nature Index for Parents of Preschool Children (CNI-PPC), the Dimensional Change Card Sorting Task (DCCS), the Devereux Early Childhood Assessment for Preschoolers, Second Edition (DECA-P2), and the Williams Creative Personality Scale for Preschoolers were used to assess connectedness to nature, cognitive flexibility, psychological resilience, and creative personality, respectively. Independent-samples *t*-tests, one-way analysis of variance (ANOVA), correlation analysis, and parallel mediation analysis were conducted to test the proposed model. Results: Connectedness to nature was significantly and positively associated with cognitive flexibility, psychological resilience, and creative personality. Cognitive flexibility and psychological resilience were both positively associated with children’s creative personality. Furthermore, cognitive flexibility and psychological resilience each played a significant mediating role in the relationship between connectedness to nature and creative personality. The association between connectedness to nature and creative personality was further explained by the parallel mediating effects of cognitive flexibility and psychological resilience. Conclusions: Connectedness to nature was positively associated with preschool children’s creative personality, both directly and indirectly through cognitive flexibility and psychological resilience. These findings provide empirical evidence for understanding the cognitive and emotional pathways linking nature connectedness and creative personality development in early childhood.

## 1. Introduction

Creative personality refers to a relatively stable and integrated set of personality traits exhibited by an individual during creative activities (Liu et al., 2022). As a fundamental component of creativity, it serves as an important driving force behind creative activities and helps individuals realize their creative potential (Dudek & Hall, 1991). The Organization for Economic Co-operation and Development (OECD) has pointed out that creativity is becoming increasingly important in today’s labor market (Vincent-Lancrin, 2019). It not only helps individuals stand out in their careers but also makes life more fulfilling and meaningful. In an era of rapid advances in artificial intelligence, creativity remains a uniquely human capacity that is difficult to replicate. It enhances individual well-being and contributes to a healthy and democratic society. The primary characteristics of creative personality include risk-taking, curiosity, imagination, and a willingness to embrace challenges. Creative personality is an important foundation for innovation and a key factor associated with creative achievement (Feist & Barron, 2003). Early childhood is a critical period for the development of creative personality. Given their stage of cognitive development, preschool children’s creativity is often expressed through creative potential and individual creative expression (Runco, 2003). Therefore, fostering young children’s creative personality and investigating its determinants are of critical importance.

In recent years, connectedness to nature, which refers to an individual’s cognitive, emotional, and behavioral relationship with the natural world, has received increasing attention as a potential factor associated with creative development in young children. In preschool children, connectedness to nature is reflected in their emotional connection to nature, interest in exploring the natural world, participation in nature-related activities, and direct experiences with natural environments (Sobko et al., 2018). Therefore, children’s connectedness to nature is likely to be shaped by both individual tendencies and environmental contexts. Studies have shown that children with higher levels of connectedness to nature tend to be more adventurous, more socially adept, and possess more positive psychological traits (Cervinka et al., 2012). Other research suggests that plants, animals, and other features of natural environments can stimulate children’s curiosity and encourage exploration, thereby supporting creativity (Norðdahl & Einarsdóttir, 2015). Early childhood represents a critical period for bonding with nature (Kahn & Kellert, 2002), and existing evidence confirms that nature connectedness positively influences preschoolers’ creative personality (J. He et al., 2024).

Recent evidence from a mixed-methods systematic review further suggests that nature-based early childhood education is positively associated with children’s cognitive, social, and emotional development (Johnstone et al., 2022). These findings highlight the developmental value of nature-related experiences and underscore the importance of examining the cognitive and emotional mechanisms associated with children’s creative personality. Previous studies have paid limited attention to the interplay between environmental and individual factors, limiting a comprehensive understanding of creative personality development in early childhood. Cognitive factors may represent an important pathway through which nature-related experiences are associated with creativity, as suggested by the extended Attention Restoration Theory model (Tang et al., 2023). The model proposes that exposure to nature broadens individuals’ cognitive scope, thereby facilitating the generation of new ideas and insights. Previous studies have shown that contact with nature is associated with improvements in attention control and executive functions, particularly working memory and cognitive flexibility. Smaller improvements have also been observed in inhibitory control following exposure to natural environments (Stevenson et al., 2018). Therefore, this study hypothesizes that the extended Attention Restoration Theory model also applies to young children: connectedness to nature may promote cognitive flexibility, thereby enhancing young children’s creative personality.

On the other hand, recent research on creative personality suggests that sustained engagement in creative activities depends not only on cognitive abilities but also on individuals’ psychological adaptability (Yao et al., 2023). This adaptability enables individuals to cope with setbacks and remain engaged when facing challenges during the creative process. Psychological resilience reflects individuals’ ability to adapt positively and recover from challenges. Previous research has shown that psychological resilience is associated with creative personality traits such as curiosity and risk-taking. For example, Caroli and Sagone (2014) found a significant positive relationship between psychological resilience and creative personality. Adolescents with higher levels of psychological resilience were more likely to exhibit creative traits such as curiosity and a willingness to take risks. Existing research suggests that natural environments may support the development of psychological resilience by reducing stress and enhancing positive psychological resources. However, findings regarding the relationship between connectedness to nature and psychological resilience in early childhood remain inconsistent. For example, Tarman et al. found that preschool children’s affinity for nature was significantly associated with the self-control dimension of psychological resilience, but not with the initiative or attachment dimensions (Tarman et al., 2023). In addition, a longitudinal intervention study found that children who participated in nature-based classes over an extended period showed greater improvement in the initiative dimension of psychological resilience. Integrating nature-based activities into traditional classrooms was also associated with positive effects on self-regulation and attachment (Ernst et al., 2021). Therefore, examining the relationship between connectedness to nature and psychological resilience in young children may help explain how nature-related experiences are associated with creative personality development.

Although previous studies have examined the relationships among connectedness to nature, cognitive flexibility, psychological resilience, and young children’s creative personality, the mechanisms linking these variables remain unclear. In particular, while connectedness to nature has been associated with creative personality, the potential mediating roles of cognitive flexibility and psychological resilience have received limited attention. Therefore, the present study investigates whether cognitive flexibility and psychological resilience mediate the relationship between connectedness to nature and young children’s creative personality.

## 2. Mediating Role of Cognitive Flexibility

Cognitive flexibility is a core component of executive function and is often described as set-shifting, attention switching, or task switching. It refers to the ability to disengage from information that is no longer relevant and adapt attention to new task demands (Monsell, 2003). It is a complex process that encompasses a range of adaptive behaviors, such as multitasking, generating novel ideas and solving problems flexibly (Ionescu, 2012). According to Attention Restoration Theory, natural environments can help individuals replenish their attentional resources (Kaplan & Kaplan, 1989). In this view, natural environments can restore depleted directed attention and support cognitive functioning. Existing research suggests that exposure to nature is associated with better executive function and self-regulation skills in young children, although evidence in early childhood remains limited (Madzia et al., 2019; Taylor et al., 2002). For example, a systematic review found that nature-based interventions may improve attention control and executive function. The strongest effects were observed for working memory and cognitive flexibility, whereas improvements in inhibitory control were more modest. (Stevenson et al., 2018). However, these findings may not fully generalize to young children because cognitive functioning and responses to nature exposure vary across developmental stages. In psychology, connectedness to nature refers to a positive relationship between individuals and the natural world (Barrable & Booth, 2020). Moreover, researchers have developed reliable and well-validated instruments to assess connectedness to nature in children. Taken together, the existing evidence suggests a positive relationship between connectedness to nature and cognitive flexibility. Therefore, we hypothesize that connectedness to nature will be positively associated with cognitive flexibility in young children.

Over recent years, creativity and executive functions (EFs) have been highly valued and sought-after constructs in society (Runco, 2015), and scientific evidence shows that EFs play an important role in adult creativity (Nusbaum & Silvia, 2011). Meanwhile, a study with children showed that, within the executive functions, task shifting was the strongest predictor of creative development (J. Wang et al., 2021). Research suggests that creativity is related to shifting ability because it requires flexibility of thought to produce new and different ideas (Bernabeu-Brotons & De la Peña, 2021; Pan & Yu, 2018). According to the dual pathway to creativity model (DPCM), one of the pathways is the flexibility route (De Dreu et al., 2008). Cognitive flexibility involves processing information across a broad range of cognitive categories and shifting between them when needed. This ability enables individuals to connect diverse concepts and generate ideas from multiple perspectives (Nijstad et al., 2010). As cognitive flexibility increases, it allows remote ideas to enter working-memory processing, thereby sparking creativity (Dreisbach & Goschke, 2004). Empirical studies (Zabelina et al., 2016) have also shown that cognitive flexibility measured with behavioral paradigms (e.g., oddball, Stroop) is positively associated with divergent thinking and creative achievement. Previous research (De Dreu et al., 2011) has also shown that individuals with higher creativity exhibit better cognitive flexibility compared to those with lower creativity. Moreover, individual-difference work by Zabelina and Robinson (2010) found that people higher in cognitive flexibility also display greater creativity. A recent review found that cognitive flexibility was positively associated with creativity in children and adolescents (Pasarín-Lavín et al., 2023). However, research on cognitive flexibility and creative personality in preschool children remains limited. Based on findings from older age groups, we hypothesize that cognitive flexibility is positively associated with creative personality in young children.

Recently, Tang et al. (2023) drew on Attention Restoration Theory (ART) to propose a model explaining how workplace exposure to nature influences employees’ creativity by broadening cognitive processing. Specifically, contact with nature may broaden individuals’ cognitive perspectives and facilitate the generation of novel ideas. Previous research has also identified cognitive flexibility as an important mechanism underlying creativity, including its mediating role in the relationship between mindfulness and creativity (Müller et al., 2016). It further confirms the feasibility of cognitive flexibility as a psychological mechanism. Although the theories and studies reviewed above have focused primarily on adults, the core principles of Attention Restoration Theory may also be relevant to young children. Early childhood is a period of rapid cognitive development and high brain plasticity. Based on the theoretical and empirical evidence reviewed above, we hypothesize that cognitive flexibility mediates the relationship between connectedness to nature and young children’s creative personality.

## 3. Mediating Role of Psychological Resilience

Psychological resilience refers to an individual’s capacity to adapt positively to adversity and changing circumstances (Herrman et al., 2011). It enables individuals to maintain psychological well-being and recover from stress, challenges, and setbacks. Studies of restorative environments have shown that simply spending time in natural settings can also lead to reduced stress or a quicker recovery from stress (Parsons et al., 1998; Ulrich et al., 1991). Empirical work also shows that individuals who feel connected to nature cope more effectively during difficult times (Seator, 2000). Researchers have also examined the relationship between connectedness to nature and psychological resilience. The findings indicated a moderate positive association between the two variables (Ingulli & Lindbloom, 2013). At the preschool level, one study examined the relationship between nature connectedness and psychological resilience. The findings indicated a significant positive association between nature connectedness and the self-control dimension of resilience (Tarman et al., 2023). A longitudinal study found that children in nature-based classrooms showed greater improvement in the initiative dimension of psychological resilience than those in hybrid or conventional classrooms. Hybrid classrooms were also associated with higher levels of self-regulation, attachment, and overall resilience. Furthermore, greater exposure to nature was linked to higher resilience scores among children (Ernst et al., 2021). In addition, a meta-analysis found that children’s psychological resilience improved significantly following participation in nature-based interventions. These findings further support the role of nature-based experiences in promoting resilience development during childhood (Fan et al., 2025).

Creativity itself is closely linked to positive factors (Hsu, 2019). Psychological resilience has been associated with personality development and broader personality traits, suggesting its potential relevance to creative personality (Altinok Dilsa Cemre et al., 2021). As a form of positive psychological capital, psychological resilience can effectively predict superior creative performance (Sweetman et al., 2011). Resilience plays a key role in fostering creativity because it enables individuals to persist in the face of the challenges, setbacks, and failures commonly encountered during the creative process (Yao et al., 2023). Creative activities often involve uncertainty, challenges, and setbacks. Psychological resilience helps individuals cope with these difficulties, maintain motivation, and remain engaged in the creative process (Y. Xu et al., 2022). Empirical studies have also shown that psychological resilience is closely linked to creativity (Y. Liang et al., 2021; Metzl, 2009). For example, individuals high in resilience are better at coping with failure, viewing difficulties as opportunities for growth, and remaining engaged in creative tasks (Li et al., 2022; Q. Wang, 2023). Tong et al. (2022) found that resilience is positively associated with creative achievement, as individuals with higher resilience are more likely to overcome challenges and continuously refine their ideas, which is precisely the process required for innovation. Caroli and Sagone (2014) found a positive association between psychological resilience and creative personality in adolescents. Higher levels of resilience were associated with greater curiosity and a stronger willingness to take risks. A study of 1711 primary school students found that higher levels of psychological resilience were associated with greater persistence in problem solving, better stress tolerance, and higher levels of creativity (Y. Xu et al., 2022). However, research on the relationship between psychological resilience and creative personality in preschool children remains limited. Based on the theoretical and empirical evidence reviewed above, we hypothesize that psychological resilience mediates the relationship between connectedness to nature and young children’s creative personality.

## 4. The Present Study

**Figure 1:**
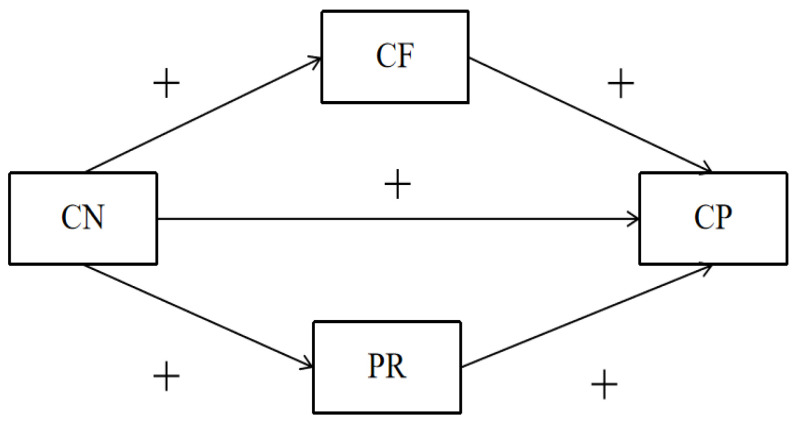
The Hypothesized Mediation Model. Note. CN, Connectedness to Nature; CF, cognitive flexibility; PR, psychological resilience, CP, Creative Personality.

Two research gaps were identified according to the literature reviewed above. First, existing research has mainly focused on the direct relationship between connectedness to nature and young children’s creative personality, with limited attention given to the role of psychological factors. According to the 4P model of creativity, creativity involves four interrelated components: Person, Process, Product, and Press (environment). This model suggests that both individual characteristics and environmental factors contribute to creative development. Second, the relationships among connectedness to nature, cognitive flexibility, psychological resilience, and creative personality in preschool children remain insufficiently understood. Although previous studies have shown that cognitive flexibility and psychological resilience may serve as important mechanisms related to creativity (X. Wang et al., 2023; W. Xu et al., 2019), most of this research has focused on university students, with relatively little attention given to preschool children. Guided by Attention Restoration Theory and the 4P model of creativity, the present study examines the relationships among connectedness to nature, cognitive flexibility, psychological resilience, and creative personality in children aged 3–6 years. It also investigates whether cognitive flexibility and psychological resilience function as parallel mediators in the relationship between connectedness to nature and creative personality (see Figure 1). The findings are expected to contribute to a better understanding of creative personality development in early childhood and provide practical implications for educational practice.

Accordingly, the following three hypotheses were proposed:

**H1.**  *Connectedness to nature is positively associated with cognitive flexibility, psychological resilience, and young children’s creative personality.*

**H2.**  *Cognitive flexibility and psychological resilience are positively associated with young children’s creative personality.*

**H3.**  *Cognitive flexibility and psychological resilience mediate the relationship between connectedness to nature and young children’s creative personality in parallel.*

## 5. Materials and Methods

### 5.1. Participants

This study was conducted in Henan Province, China, to examine the relationships among connectedness to nature, cognitive flexibility, psychological resilience, and creative personality in preschool children. To enhance sample diversity, two cities with different levels of economic development were selected based on the 2024 GDP rankings of cities in Henan Province (National Economic Accounting, 2024). N City represented a relatively more economically developed area, whereas K City represented a less economically developed area. A cluster sampling method was then adopted. One representative kindergarten was selected from each city, and questionnaires were distributed to parents through the participating kindergartens. A total of 675 questionnaires were distributed, and 652 were returned, resulting in a response rate of 96.6%. After data screening, 60 invalid questionnaires were excluded, leaving 592 valid responses and a valid response rate of 90.8%. All study procedures complied with the ethical standards of the Henan Key Laboratory of Psychology and Behavior. Before data collection, participants were informed about the purpose of the study, the survey procedures, and data confidentiality. Written informed consent was obtained from all participants. The demographic characteristics of the participants are presented in Table 1.

### 5.2. Measures

#### 5.2.1. Connectedness to Nature Index for Parents of Preschool Children (CNI-PPC)

In this study, we employed the Chinese version of the Connectedness to Nature Index for Parents of Preschool Children (CNI-PPC), developed by Sobko et al. (2018), to assess the level of connectedness to nature in preschool children. This instrument comprises four dimensions: Enjoyment of Nature (six items), which refers to a child’s liking for interacting with nature and feeling comfortable and happy in natural settings, such as “enjoys listening to the different sounds of nature”; Empathy for Nature (three items), indicating a child’s emotional response of unhappiness or sadness when animals or plants are hurt or die, like “feels upset when animals are hurt”; Responsibility toward Nature (three items), denoting a child’s protective behaviors towards the environment, such as “takes care of plants and animals”; and Awareness of Nature (four items), representing a child’s perception or recognition of the natural world in various ways, for example, “notices wildlife wherever he/she is.” The scale consists of 16 items in total, scored on a 5-point Likert scale ranging from “disagree” to “agree.” Higher scores indicate a stronger connection to nature. In this study, the overall Cronbach’s alphas were 0.905, and Cronbach’s alphas for the dimensions of enjoyment of nature, empathy for nature, responsibility toward nature, and awareness of nature were 0.850, 0.841, 0.680, and 0.779.

#### 5.2.2. The Williams Creative Personality Scale for Preschoolers

The Williams scale, originally developed by Williams and later revised by Lin and Wang (1994), is a creative personality test that can be used by parents and teachers to assess children’s creative personality traits. It comprises 50 items grouped into four subscales: adventurousness, curiosity, imagination, and challenge. All items are rated on a 3-point scale (‘does not apply at all,’ ‘partially applies’, ‘fully applies’); higher scores indicate more pronounced creative personality traits in young children. Subsequently, Chinese scholars Gao et al. (2021) further refined the scale. Ten items whose item-total correlations were below r < 0.30 were removed, and several statements were reworded to better suit the kindergarten context. The resulting 40-item scale demonstrated high internal consistency (Cronbach’s α = 0.946) and excellent model fit: χ^2^/df = 4.60, CFI = 0.985, TLI = 0.985, RMSEA = 0.037, SRMR = 0.038. The revised scale retains the following four dimensions: (1) Challenge (e.g., ‘likes to investigate whether things are true or false’); (2) Imagination (e.g., ‘likes to fantasize about future life situations’); (3) Curiosity (e.g., ‘likes things that are different from the ordinary’); and (4) adventurousness (e.g., ‘likes toys that can be taken apart’). This revised version has been validated in China as appropriate for parental assessment of preschoolers’ creative personality. In this study, the overall Cronbach’s alphas were 0.921, and Cronbach’s alphas for the dimensions of consistency of challenge, imagination, curiosity and adventurousness were 0.782, 0.704, 0.803 and 0.830.

#### 5.2.3. Dimensional Change Card Sorting Task (DCCS)

The Dimensional Change Card Sorting (DCCS) task, developed by Zelazo (2006), was used to measure the cognitive flexibility of preschool children. In the task, the experimenter presented children with cards that varied along two dimensions—color and shape. First, the child had to sort six cards by color; after that, he or she was asked to resort six new cards by shape (the order of the two rules was counter-balanced across participants, the first being the pre-switch and the second the post-switch phase). Six cards were used per dimension, and five correct sorts within a phase were required to pass. When both standard phases were finished, the child moved on to the border version: cards now had to be classified by color if they bore a black border, or by shape if they did not. This block comprised 12 trials, with nine correct classifications needed to pass. Cognitive-flexibility scores were assigned as follows: 0 = failed the pre-switch phase; 1 = passed pre-switch but failed post-switch; 2 = passed both standard phases but failed the border block; 3 = passed all three parts (pre-switch, post-switch, and border).

#### 5.2.4. Devereux Early Childhood Assessment for Preschoolers Second Edition (DECA-P2)

The Devereux Early Childhood Assessment for Preschoolers, Second Edition (DECA-P2), is a behavior rating scale completed by parents and/or teachers to assess protective factors related to social-emotional development and psychological resilience in children aged 3–5 years. The scale also serves as a screening tool for behavioral concerns. It consists of 38 items designed to measure the frequency of the described behaviors exhibited by children aged 3–5 years over the past four weeks, including subscales: Initiative (IN), Self-Regulation (SR), Attachment/Relationship (AR), and Behavioral Concerns (BC). The Chinese translation of the scale was ultimately adopted for formal distribution in this study. There is a total of 21 items (S. H. Liang et al., 2019). Initiative (9 items) measures the child’s ability to use independent thought and action to meet his/her needs. Self-regulation (6 items) measures the child’s ability to express emotions and manage behaviors in healthy ways. Attachment/Relationship (6 items) measures the child’s ability to promote and maintain mutual, positive connections with other children and significant adults. The raw scores from the three subscales—initiative, self-regulation, and attachment/relationship—are converted to T-scores and then summed to obtain the Total Protective Factors (TPF) subscale score. T = 50 + 10z, where z is the standard score. In the original scale, z values were calculated based on international normative parameters, while in this study, z values were calculated based on sample parameters. In this study, the overall Cronbach’s alphas were 0.865, and Cronbach’s alphas for the dimensions of initiative, self-regulation, attachment/relationship were 0.792, 0.745, and 0.667).

### 5.3. Procedures

Informed consent forms were distributed to parents, who were invited to sign them if they agreed to participate. Questionnaires were then distributed through homeroom teachers. One parent from each family completed the Connectedness to Nature Index for Parents of Preschool Children (CNI-PPC), the Devereux Early Childhood Assessment for Preschoolers, Second Edition (DECA-P2), and the Williams Creative Personality Scale for Preschoolers. Executive function assessments were administered individually by trained graduate and undergraduate students majoring in early childhood education. Before data collection, all examiners received standardized training to ensure a consistent understanding of executive function assessment procedures and task administration requirements. Assessments were conducted individually in a quiet, spacious, and familiar room within the kindergarten. Before each task, the examiner explained the instructions and verified that the child understood the task requirements. Formal testing began only after the child demonstrated a correct understanding of the task. Upon completion of the assessment, each child received a small gift as a token of appreciation.

### 5.4. Ethical Consideration

The study was approved by the Ethics Committee of the first author’s university and the Henan Provincial Key Laboratory of Psychology and Behavior (Approval No. 20241008002). All procedures were conducted in accordance with the ethical principles of the Declaration of Helsinki. Written informed consent was obtained from all participants prior to data collection. Participation was voluntary, and participants were informed of their right to withdraw from the study at any time without penalty. All questionnaire data were collected anonymously and coded to protect participants’ privacy. Research assistants received standardized training and were required to maintain strict confidentiality throughout the data collection process.

### 5.5. Analytic Approach

The current study used SPSS 19.0 to preprocess the data to obtain descriptive statistics and bivariate correlation results. One structural equation model was used to test the possible mediating effects of cognitive flexibility and psychological resilience on the association between connectedness to nature and young children’s creative personality, using AMOS 28.0. The conceptual and theoretical model for such analysis is the parallel multiple mediator model, in which the antecedent variable X (Connectedness to Nature in the current study) is modeled as influencing the consequent variable Y (children’s creative personality) both directly and indirectly through two or more mediators. Including two or more mediators in an integrated model allows for a formal comparison of the unique indirect effects of X via each of them. Bias-corrected bootstrap method was performed to evaluate the significance of the mediated paths. The Bootstrap method was employed, specifically a bias-corrected bootstrap with 5000 resamples, to compute 95% confidence intervals (CIs) and test the mediating roles of cognitive flexibility and psychological resilience.

### 5.6. Common Method Biases

To assess the potential influence of common method bias, Harman’s single-factor test was conducted. An unrotated exploratory factor analysis revealed 19 factors with eigenvalues greater than 1. The first factor accounted for 19.901% of the total variance, which was well below the recommended threshold of 40%. Therefore, common method bias was not considered a serious concern in the present study, and subsequent statistical analyses were deemed appropriate.

## 6. Results

### 6.1. Descriptive Statistics

The descriptive statistics of the key variables and the results of ANOVA and independent-samples *t*-tests are shown in Table 2. The results showed that connectedness to nature differed significantly according to place of residence, gender, only-child status, parental educational background, and age. Cognitive flexibility was significantly associated with place of residence, parental educational background, and age. Psychological resilience varied significantly across parental educational background and age groups. In contrast, family structure was not significantly related to any of the study variables. For creative personality, a significant age effect was observed, whereas no significant differences were found across place of residence, gender, only-child status, parental educational background, or family structure. To understand the relationship and development of variables, Spearman correlation analysis was carried out (see Table 3). The results showed connectedness to nature, cognitive flexibility, psychological resilience, and young children’s creative personality were all significantly inter-correlated. The results of the relational analysis verified Hypothesis 1.

### 6.2. The Mediating Roles of Cognitive Flexibility and Psychological Resilience

**Figure 2:**
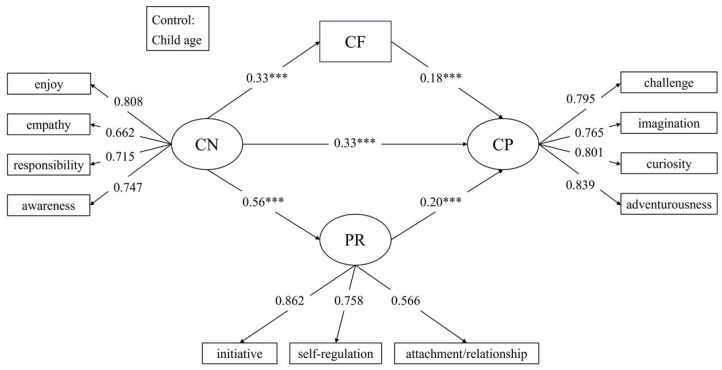
The parallel Mediating Model Diagram. Note. CN, connectedness to nature; CF, cognitive flexibility; PR, psychological resilience, CP, creative personality. *** *p* < 0.001.

To examine whether cognitive flexibility and psychological resilience mediate the pathway from nature connectedness to young children’s creative personality, we conducted a mediational SEM while controlling for child age. The two mediators (cognitive flexibility and psychological resilience) were entered simultaneously into a single model. Results showed that the model provided an excellent fit to the data: χ^2^/df = 3.562, between 1 and 5; GFI = 0.947, AGFI = 0.919, IFI and CFI = 0.949, both greater than 0.9; RMSEA = 0.066, less than 0.08, close to 0.07. The results of path analysis were significant (see Figure 2). Connectedness to nature exerts a significantly positive effect on creative personality (*β* = 0.33, *p* < 0.001). The path from connectedness to nature to cognitive flexibility had a positive and significant effect (*β* = 0.33, *p* < 0.001), and the path from cognitive flexibility to creative personality was also positively and significantly influenced (*β* = 0.18, *p* < 0.001). Meanwhile, connectedness to nature also positively predicted psychological resilience (*β* = 0.56, *p* < 0.001), and psychological resilience, in turn, positively predicted creative personality (*β* = 0.20, *p* < 0.001).

The mediating effects of cognitive flexibility and psychological resilience are detailed in Table 4. The standardized direct effect of connectedness to nature on young children’s creative personality was 0.330 (SE = 0.058, 95% CI = 0.206 to 0.436). The indirect path from connectedness to nature to creative personality through cognitive flexibility was significant, with an effect of 0.059 (SE = 0.016, 95% CI = 0.033 to 0.099), accounting for 34.91% of the total indirect effect. The indirect path through psychological resilience was also significant, with an effect of 0.110 (SE = 0.036, 95% CI = 0.043 to 0.185), accounting for 65.09% of the total indirect effect. The total indirect effect of cognitive flexibility and psychological resilience was 0.169 (SE = 0.038, 95% CI = 0.101 to 0.255), confirming the significance of the overall mediation effect. As none of the bootstrap 95% confidence intervals included zero, all indirect effects were considered statistically significant. These results support the parallel mediation model proposed in this study, suggesting that connectedness to nature may indirectly affect the development of young children’s creative personality through cognitive flexibility and psychological resilience.

## 7. Discussion

In this study, we examined the parallel mediating roles of cognitive flexibility and psychological resilience in the relationship between connectedness to nature and young children’s creative personality. Significant positive associations were found among connectedness to nature, cognitive flexibility, psychological resilience, and creative personality. Furthermore, cognitive flexibility and psychological resilience jointly and partially mediated the relationship between connectedness to nature and creative personality. These results indicate that cognitive flexibility and psychological resilience may serve as two important psychological mechanisms linking connectedness to nature and creative personality. The present study extends previous research by demonstrating that connectedness to nature is associated not only with children’s creative personality, but also with their cognitive flexibility and psychological resilience. Specifically, children with higher levels of connectedness to nature tended to report greater cognitive flexibility and psychological resilience, which may help explain the positive relationship between connectedness to nature and creative personality.

### 7.1. The Mediating Roles of Young Children’s Cognitive Flexibility

The present study found that cognitive flexibility partially mediated the relationship between connectedness to nature and young children’s creative personality. Specifically, children with stronger connectedness to nature tended to exhibit greater cognitive flexibility, which was in turn associated with higher levels of creative personality. Previous studies have shown that exposure to natural environments can improve working memory, cognitive flexibility, and inhibitory control (Stevenson et al., 2018). According to the dual-pathway model of creativity, the development of individual creativity requires the involvement of cognitive flexibility, and numerous studies have demonstrated that individuals with higher cognitive flexibility also exhibit greater creativity (De Dreu et al., 2011; Zabelina & Robinson, 2010). One possible explanation is that cognitive flexibility enables individuals to process information through broader cognitive categories and to shift efficiently between different perspectives and strategies. Such flexibility may help individuals overcome habitual ways of thinking, maintain a wider attentional focus, and form novel associations, thereby facilitating creative thinking and creative personality development (Nijstad et al., 2010). However, this relationship has rarely been examined in young children. Our findings indicate that preschoolers’ cognitive flexibility is positively associated with creative personality, extending previous research by demonstrating this relationship in a younger age group.

As Tang et al. (2023) found with their ART-based model, exposure to nature may be associated with broader cognitive processing, which may facilitate creativity: nature redirects attention from the immediate setting to a broader environmental scope, widening mental horizons and facilitating novel ideas. Furthermore, connectedness to nature may provide important conditions for the development of cognitive flexibility during early childhood. Interactions with natural environments often involve novelty, variability, and open-ended exploration, requiring children to continuously adjust their attention and behavior in response to changing situations. Such experiences may promote the development of more flexible cognitive patterns. Children with higher levels of cognitive flexibility are more likely to approach creative activities from different perspectives, explore multiple possibilities, and respond to challenges in adaptive ways. These characteristics may contribute to the development of creative personality. The present findings provide support for the relevance of Attention Restoration Theory in early childhood and highlight the important roles of connectedness to nature and cognitive flexibility in creative personality development. As a mediating factor, cognitive flexibility may help explain how connectedness to nature is associated with children’s creative personality. Specifically, nature-related experiences may broaden children’s cognitive perspectives, support sustained attention, and encourage flexible responses to different situations, thereby contributing to creative personality development.

### 7.2. The Mediating Roles of Young Children’s Psychological Resilience

Connectedness to nature was positively associated with young children’s psychological resilience, which is consistent with the findings of Ingulli and Lindbloom (2013). Studies of restorative environments have shown that simply spending time in natural settings can also lead to reduced stress or a quicker recovery from stress (Parsons et al., 1998; Ulrich et al., 1991). Previous research has shown that individuals who feel connected to nature cope more effectively during difficult times (Seator, 2000). One possible explanation is that children with higher levels of connectedness to nature are more likely to experience positive emotions and gain psychological resources through interactions with natural environments. Nature often provides safe, relaxing, and supportive settings that help children maintain positive psychological states. These experiences may contribute to the development of psychological resilience. Such sustained positive emotional experiences help children develop stronger emotional regulation capabilities and adaptability when facing stress, difficulties, or failures, which may contribute to higher levels of psychological resilience of psychological resilience. The study also confirms a significant positive association between young children’s psychological resilience and creative personality, consistent with the results reported by Caroli and Sagone (2014). Psychological resilience may play an important role in children’s creative development by helping them cope with challenges, persist in the face of setbacks, and remain engaged in creative activities (Yao et al., 2023). It enables individuals to recover from setbacks, maintain motivation, and continuously seek novel solutions (Y. Xu et al., 2022).

Moreover, highly resilient individuals are better at coping with failure and can sustain their engagement in creative tasks (Li et al., 2022). The emergence of this relationship may be related to the shared neural basis between creativity and psychological resilience. A recent study using ultra-high-field magnetic resonance imaging revealed the neurobiological commonalities of psychological resilience and the Big Five personality traits at the level of three core resting-state networks (the DMN, SN, and the central executive network) (Altinok Dilsa Cemre et al., 2021). Studies have shown that functional connectivity within the DMN is associated with psychological resilience in healthy participants at rest (L. He et al., 2018). Patients with PTSD who exhibit low levels of psychological resilience demonstrate impaired static functional connectivity in DMN subsystems (Miller et al., 2017).

While the existing literature has highlighted resilience as an important factor in creative development (Li et al., 2022; Q. Wang, 2023). However, most previous research has focused on adolescents and adults. The present findings suggest that psychological resilience may be an important mechanism linking connectedness to nature and creative personality in young children. Specifically, psychological resilience may help translate the positive emotional experiences and psychological resources associated with connectedness to nature into characteristics that support creative personality development.

### 7.3. The Parallel Mediating Roles of Young Children’s Cognitive Flexibility and Psychological Resilience

The present study highlights the parallel mediating roles of cognitive flexibility and psychological resilience in the relationship between connectedness to nature and young children’s creative personality. The findings suggest that connectedness to nature is associated with children’s creative personality through both cognitive and emotional pathways. By integrating cognitive flexibility and psychological resilience into a single model, the present study provides a more comprehensive understanding of the psychological processes underlying creative personality development in early childhood. These findings extend previous research by demonstrating the importance of cognitive flexibility and psychological resilience in understanding creative personality development during early childhood. The pathway of cognitive flexibility opens the possibility of achieving creative insight (Amabile, 1983). Because creativity involves “breaking the routine” and overcoming “functional fixedness” (X. Wang et al., 2018). Creative activities often require individuals to move beyond habitual patterns of thinking, broaden their attentional focus, and flexibly consider alternative ideas and solutions. Cognitive flexibility may therefore play an important role in creative personality development. Psychological resilience, in contrast, helps individuals tolerate uncertainty, cope with setbacks, and remain engaged in creative activities despite difficulties. These characteristics are particularly important for sustaining creativity throughout the iterative and often challenging creative process (Y. Xu et al., 2022). By promoting cognitive flexibility and psychological resilience, connectedness to nature may support the development of young children’s creative personality. Notably, although both variables partially mediated the relationship between connectedness to nature and creative personality, the indirect effect of psychological resilience was stronger than that of cognitive flexibility. This finding suggests that emotional adaptation may play a more important role than cognitive factors in explaining the association between connectedness to nature and creative personality during early childhood.

### 7.4. Limitations and Directions for Future Research

Some limitations of this study should be addressed in future research.

First, the participants were recruited from only two kindergartens within Henan Province, which may limit the representativeness of the sample and the generalizability of the findings. Future research should include participants from different regions and socioeconomic backgrounds in China to enhance the external validity of the results.

Second, this study employed a cross-sectional design and collected data at a single point in time, similar to other empirical studies (J. He et al., 2024). Therefore, the findings should be interpreted as associations among variables rather than evidence of causal relationships. Although the proposed mediation model was statistically supported, the present design does not allow causal inferences regarding the direction of the observed effects. Future studies could adopt cross-lagged panel designs, longitudinal approaches, or experimental methods to further examine the causal relationships among connectedness to nature, cognitive flexibility, psychological resilience, and creative personality.

Finally, the present study relied primarily on quantitative measures. Future research may benefit from incorporating qualitative or mixed-method approaches, such as children’s drawings, interviews, and observational methods, to gain a richer understanding of how children perceive and experience their relationship with nature.

### 7.5. Theoretical and Practical Contributions

Theoretically, this study extends the application of Attention Restoration Theory to early childhood development by suggesting that the benefits of nature may not be limited to attentional restoration but may also be reflected in broader cognitive characteristics related to creativity. Furthermore, from the perspective of the 4P Model of Creativity, the findings provide empirical support for the role of environmental factors in creative personality development and contribute to a better understanding of how environmental, cognitive, and emotional factors jointly relate to creativity in early childhood.

From an educational perspective, the findings suggest that kindergartens and families should provide children with more opportunities to engage with natural environments through outdoor exploration, nature-based learning activities, gardening projects, and unstructured play in natural settings. Such experiences may help strengthen children’s connectedness to nature while simultaneously supporting the development of cognitive flexibility and psychological resilience. Recent research on outdoor learning in early childhood education has similarly emphasized that nature-based experiences can support children’s holistic development, wellbeing, and learning opportunities, further highlighting the educational value of providing regular opportunities for engagement with natural environments (Leena et al., 2024). Furthermore, recent intervention research has suggested that the developmental benefits of outdoor environments are enhanced when teachers intentionally integrate nature-based experiences into educational practice (Berg et al., 2024). Specifically, educators can implement open-ended nature exploration, imaginative play, and problem-solving activities in natural settings to encourage children to observe, reflect, and explore multiple solutions, thereby promoting the development of cognitive flexibility. At the same time, appropriately challenging outdoor activities, such as obstacle-based games, cooperative adventure tasks, and nature-based adventure play, may help foster persistence, emotional regulation, and psychological resilience. Previous research has shown that challenging outdoor activities and nature-based adventure experiences can promote children’s adaptive functioning, self-regulation, and psychological resilience (Brussoni et al., 2015). Therefore, educational practices should not only increase children’s opportunities to engage with nature but also support the development of cognitive flexibility and psychological resilience, which may contribute to creative personality development.

## 8. Conclusions

This study found that connectedness to nature was positively associated with preschool children’s creative personality. Cognitive flexibility and psychological resilience each served as independent mediators in the relationship between connectedness to nature and preschool children’s creative personality, and together constituted a parallel mediation effect.

## Figures and Tables

**Table 1:** Demographic characteristics of the participants in the study (*N* = 592).

Participant	*N* (%)
Place of residence	
N City	331 (55.9)
K City	261 (44.1)
Gender of preschool children	
Boy	314 (53)
Girl	278 (47)
Only child	
Yes	169 (28.5)
No	423 (71.5)
Father’s educational level	
Junior high school and below	48 (8.1)
Technical secondary school or high school	86 (14.5)
Junior college	150 (25.3)
Undergraduate	284 (48)
Postgraduate and above	24 (4.1)
Mother’s educational level	
Junior high school and below	35 (5.9)
Technical secondary school or high school	89 (15)
Junior college	142 (24)
Undergraduate	286 (48.3)
Postgraduate and above	40 (6.8)
Age of preschool children	
3–4 (including 4)	127 (21.5)
4–5 (including 5)	173 (29.2)
5–6 (including 6)	198 (33.4)
6 years old and above	94 (15.9)

**Table 2:** Descriptive Statistics and ANOVA and Independent-Samples *t*-Test Results for Demographic Characteristics.

Participant Characteristics		CN	CF	PR	CP
Place of residence	K City	4.07 ± 0.52	4.45 ± 0.51	2.86 ± 0.42	1.34 ± 0.33
	N City	3.92 ± 0.52	4.29 ± 0.46	2.79 ± 0.46	1.36 ± 0.29
	t	3.34 ***	3.92 ***	1.86	−0.92
Gender of preschool children	Boy	3.94 ± 0.50	4.35 ± 0.48	2.80 ± 0.45	1.34 ± 0.30
	Girl	4.05 ± 0.55	4.38 ± 0.49	2.84 ± 0.44	1.37 ± 0.31
	t	−2.41 *	−0.76	−0.99	−1.17
Only child	Yes	4.09 ± 0.48	4.40 ± 0.49	2.84 ± 0.39	1.37 ± 0.30
	No	3.95 ± 0.54	4.35 ± 0.48	2.81 ± 0.46	1.35 ± 0.31
	t	2.92 **	1.29	0.86	0.87
Parental educational background	① Junior high school and below	3.85 ± 0.60	4.20 ± 0.41	2.64 ± 0.56	1.37 ± 0.33
	② Technical secondary school or high school	3.84 ± 0.45	4.26 ± 0.44	2.73 ± 0.45	1.32 ± 0.28
	③ Junior college	3.92 ± 0.53	4.35 ± 0.48	2.80 ± 0.48	1.35 ± 0.33
	④ Undergraduate	4.07 ± 0.52	4.41 ± 0.50	2.88 ± 0.40	1.35 ± 0.30
	⑤ Postgraduate and above	4.17 ± 0.48	4.45 ± 0.50	2.81 ± 0.41	1.46 ± 0.30
	F	6.27 ***	2.96 *	3.67 **	1.62
Family structure	① Nuclear family	4.06 ± 0.53	4.39 ± 0.50	2.86 ± 0.45	1.37 ± 0.32
	② Stem family	3.93 ± 0.51	4.32 ± 0.47	2.77 ± 0.44	1.33 ± 0.29
	③ Joint family	3.56 ± 0.33	4.50 ± 0.58	2.58 ± 0.13	1.33 ± 0.31
	④ Single-parent family	3.86 ± 0.54	4.22 ± 0.44	2.89 ± 0.29	1.40 ± 0.21
	⑤ Blended family	3.68 ± 0.42	4.40 ± 0.55	2.91 ± 0.41	1.18 ± 0.29
	F	3.25 *	1.05	1.68	0.98
Age of preschool children	① 3–4 (including 4)	3.88 ± 0.50	4.10 ± 0.30	2.71 ± 0.48	1.26 ± 0.28
	② 4–5 (including 5)	4.03 ± 0.51	4.28 ± 0.46	2.83 ± 0.46	1.32 ± 0.32
	③ 5–6 (including 6)	4.03 ± 0.56	4.49 ± 0.50	2.85 ± 0.42	1.40 ± 0.30
	④ 6 years old and above	4.01 ± 0.50	4.59 ± 0.50	2.87 ± 0.41	1.43 ± 0.28
	F	2.74 *	29.20 ***	3.46 *	7.95 ***

Note. CN, Connectedness to Nature; CF, cognitive flexibility; PR, psychological resilience, CP, Creative Personality. * *p* < 0.05, ** *p* < 0.01, *** *p* < 0.001.

**Table 3:** Correlation Analysis among the Key Variables (*N* = 592).

Variables	1	2	3	4
1. Connectedness to Nature	1			
2. Cognitive Flexibility	0.287 **	1		
3. Psychological Resilience	0.461 **	0.242 **	1	
4. Creative Personality	0.427 **	0.332 **	0.357 **	1
Mean	3.99	4.36	2.82	1.35
SD	0.53	0.48	0.44	0.31

Note. ** *p* < 0.01.

**Table 4:** Mediating effects of cognitive flexibility and psychological resilience between the connectedness to nature and creative personality.

Paths	Effect	SE	BC 95% CI		Effect Size
			LL	UL	
CN → CF → CP	0.059	0.016	0.033	0.099	34.91%
CN → PR → CP	0.110	0.036	0.043	0.185	65.09%
CN → CP	0.330	0.058	0.206	0.436	
Total mediating effect	0.169	0.038	0.101	0.255	100.00%
Total effect	0.499	0.040	0.417	0.516	

## Data Availability

The original contributions presented in this study are included in the article. Further inquiries can be directed to the corresponding author.
